# Long-Term Surgical Outcomes of Liver Resection for Hepatocellular Carcinoma in Patients With HBV and HCV Co-Infection: *A Multicenter Observational Study*

**DOI:** 10.3389/fonc.2021.700228

**Published:** 2021-07-29

**Authors:** Hang-Dong Jia, Lei Liang, Chao Li, Han Wu, Hong Wang, Ying-Jian Liang, Ya-Hao Zhou, Wei-Min Gu, Xin-Ping Fan, Wan-Guang Zhang, Ting-Hao Chen, Zhi-Yu Chen, Jian-Hong Zhong, Wan Yee Lau, Timothy M. Pawlik, Yong-Kang Diao, Qiu-Ran Xu, Feng Shen, Cheng-Wu Zhang, Dong-Sheng Huang, Tian Yang

**Affiliations:** ^1^Department of Hepatobiliary Pancreatic and Minimal Invasive Surgery, Zhejiang Provincial People’s Hospital (People’s Hospital of Hangzhou Medical College), Hangzhou, China; ^2^School of Clinical Medicine, Hangzhou Medical College, Hangzhou, China; ^3^Department of Hepatobiliary Surgery, Eastern Hepatobiliary Surgery Hospital, Second Military Medical University (Navy Medical University), Shanghai, China; ^4^Department of General Surgery, Liuyang People’s Hospital, Hunan, China; ^5^Department of Hepatobiliary Surgery, The First Affiliated Hospital of Harbin Medical University, Heilongjiang, China; ^6^Department of Hepatobiliary Surgery, Pu’er People’s Hospital, Yunnan, China; ^7^The First Department of General Surgery, The Fourth Hospital of Harbin, Heilongjiang, China; ^8^Department of General Surgery, Pingxiang Mining Group General Hospital, Jiangxi, China; ^9^Department of Hepatic Surgery, Tongji Hospital, Huazhong University of Science and Technology, Wuhan, China; ^10^Department of General Surgery, Ziyang First People’s Hospital, Sichuan, China; ^11^Department of Hepatobiliary Surgery, Southwest Hospital, Third Military Medical University (Army Medical University), Chongqing, China; ^12^Department of Hepatobiliary Surgery, Affiliated Tumor Hospital of Guangxi Medical University, Nanning, China; ^13^Faculty of Medicine, The Chinese University of Hong Kong, Hong Kong, China; ^14^Department of Surgery, Ohio State University, Wexner Medical Center, Columbus, OH, United States

**Keywords:** hepatocellular carcinoma, hepatectomy, hepatitis B virus, hepatitis C virus, overall survival, recurrence-free survival

## Abstract

**Background:**

Hepatocellular carcinoma (HCC) is one of the most serious consequences of chronic hepatitis B virus (HBV) or hepatitis C virus (HCV) infection. This study sought to investigate long-term outcomes after liver resection for HCC among patients with HBV/HCV co-infection (HBV/HCV-HCC) compared with patients with HBV infection (HBV-HCC).

**Methods:**

Patients who underwent curative-intent liver resection for HCC were identified from a multicenter Chinese database. Using propensity score matching (PSM), patients with HBV/HCV-HCC were matched one-to-one to patients with HBV-HCC. Overall survival (OS) and recurrence-free survival (RFS) were compared between the two groups before and after PSM.

**Results:**

Among 2,467 patients identified, 93 (3.8%) and 2,374 (96.2%) patients had HBV/HCV-HCC and HBV-HCC, respectively. Compared with patients with HBV-HCC, patients with HBV/HCV-HCC were older, have poorer liver-related characteristics but better tumor-related characteristics. PSM created 88 pairs of patients with comparable liver- and tumor-related characteristics (all *P* > 0.2). In the PSM cohort, the 3- and 5-year RFS rates in patients with HBV/HCV-HCC were 48.3% and 38.9%, which were significantly poorer than patients with HBV-HCC (61.8% and 49.2%, *P* = 0.037). Meanwhile, the 3- and 5-year OS rates in patients with HBV/HCV-HCC were also poorer than patients with HBV-HCC (65.4% and 51.1% *vs.* 73.7% and 63.0%), with a difference close to be significant between them (*P* = 0.081).

**Conclusion:**

Comparing to patients with HBV-HCC, liver resection resulted in relatively poorer long-term surgical outcomes in patients with HBV/HCV-HCC.

## Introduction

Approximately 700,000 persons die of hepatocellular carcinoma (HCC) every year worldwide making it the third leading cause of cancer death (1, 2). Cirrhosis of the liver is the biggest risk factor associated with developing HCC (3). Overall, up to 80% of HCC cases are attributable to cirrhosis secondary to persistent viral infection, mainly hepatitis B virus (HBV) and/or hepatitis C virus (HCV) infection (4, 5). In fact, concurrent infection of HBV and HCV is not uncommon, especially in areas where both viruses are highly prevalent as the mode of transmission is the same (6, 7). Co-infection with HBV and HCV has been observed in China, Spain, Italy, Japan, and Iran with a reported prevalence of 5~40%, while 2~25% of patients with chronic HCV infection are HBsAg-positive (7). HBV-HCC accounts for approximately 75~80% of patients with HCC in China, and the proportion of Chinese patients with HCV-associated HCC has rapidly increased over the last two decades. The proportion of HCC patients with HBV/HCV co-infection has, however, likely been underestimated in China (8).

Liver resection is widely accepted as first-line curative treatment for HCC (9). Numerous studies have reported long-term outcomes after curative liver resection for patients with HBV-HCC or HCV-HCC. In virtually all previous studies, patients with HCC and HBV/HCV co-infection (i.e., HBV/HCV-HCC) were excluded from the analytic cohorts due to small case numbers. In fact, to our knowledge, there have been no studies which focused on patients with HBV/HCV-HCC. As such, the long-term surgical outcomes of patients with HBV/HCV-HCC *versus* HBV-HCC who underwent hepatic resection were compared in this study using a large multicenter database.

## Material and Methods

### Study Population

Data from a Chinese multi-institutional database of patients who underwent curative-intent liver resection for HCC between January 2005 and December 2017 at 11 hospitals were retrospectively analyzed (Zhejiang Provincial People’s Hospital, Eastern Hepatobiliary Surgery Hospital, Liuyang People’s Hospital, First Affiliated Hospital of Harbin Medical University, Pu’er People’s Hospital, Fourth Hospital of Harbin, Pingxiang Mining Group General Hospital, Tongji Hospital, Ziyang First People’s Hospital, Southwest Hospital, and Affiliated Tumor Hospital of Guangxi Medical University). The diagnosis of HCC was confirmed by histopathological examination of resected specimens. Patients less than 18 years old, as well as individuals with recurrent HCC or who underwent antitumor treatment before surgery were excluded. Curative-intent liver resection was an R0 resection, which was defined as complete resection of all microscopic and macroscopic tumors. Patients who had microscopically positive (R1 resection) or grossly positive (R2 resection) margins were excluded. As the primary aim of the study was to compare surgical outcomes between patients with HBV/HCV-HCC and HBV-related HCC, patients with HCV-HCC or with non-HBV/HCV-HCC were also excluded.

Data were collected using a standardized form both in a prospective fashion and retrospective fashion depending on the data field. The process of pre-operative work-up and evaluation among participating hospitals was virtually identical. The selection criteria for liver resection for HCC were constant over the study period and included location and number of tumors, liver functional reserve and volume of future liver remnant as reported previously (10, 11). The study was performed in accordance with the Declaration of Helsinki and was approved by the Institutional Review Boards of all the participating hospitals. Individual consent for this retrospective analysis was waived.

### Clinical Characteristics, Operative Variables, and Short-Term Outcomes

Patient demographic characteristics included age, sex, diabetes mellitus, body mass index (BMI), and American Society of Anesthesiologists (ASA) score. The clinicopathologic characteristics included Child-Pugh grading, presence/absence of cirrhosis and/or portal hypertension, serum anti-HCV antibody (anti-HCV) positivity, serum hepatitis B surface antigen (HBsAg) positivity, preoperative anti-HBV therapy, preoperative HBV-DNA level, preoperative bilirubin and albumin levels, preoperative alanine aminotransferase (ALT) and aspartate aminotransferase (AST) levels, preoperative alpha-fetoprotein (AFP) level, largest tumor size, tumor number, macroscopic or microscopic vascular invasion, satellites nodules, tumor differentiation, and tumor encapsulation were recorded. Cirrhosis was diagnosed by histopathological examination, and portal hypertension was defined as presence of either esophageal varices, or splenomegaly with a decrease in platelet count (≤ 100×10^9^/L). Operative variables included intraoperative blood loss, requirement of blood transfusion, extent of hepatectomy (major or minor), type of liver resection (anatomical or non-anatomical), and resection margin. Major hepatectomy was defined as resection of three or more Couinaud liver segments, and minor hepatectomy as resection of fewer than three segments. Anatomical resection was defined using the Brisbane 2000 Nomenclature of Liver Anatomy and Resections, while non-anatomical resections included wedge resection or limited resection (12). Short-term surgical outcomes included postoperative 30-day mortality and morbidity. Postoperative morbidity included the occurrence of postoperative acute hepatic failure, biliary complications, sepsis, pulmonary, renal, cardiac and wound complications within 30 days of surgery (11, 13, 14). Morbidity was graded according to the Clavien-Dindo classification, and major morbidity was defined as Clavien-Dindo grade ≥ 3 (15).

### Patient Follow-up

After hospital discharge, patients were followed-up in each hospital according to a relatively standardized recurrence monitoring program. In the first 6 months of operation, patients were monitored once every 1~2 months, then once every 2-3 months for the next 18 months, and thereafter once every 3~6 months (10). Patients were monitored for recurrence using physical examination, serum AFP, ultrasound or CT or MRI. When recurrence or distant metastasis was suspected, additional investigations were performed, including angiography, bone scan or positron emission tomography. Tumor recurrence was defined as emergence of intrahepatic or extrahepatic nodules with typical imaging features consistent with HCC on enhanced CT or MRI imaging regardless of serum AFP levels. Management of recurrence was based on patterns of recurrent tumors, residual liver functional reserve, and general condition of patients. Treatment included re-resection, local ablation therapy, liver transplantation, transcatheter arterial chemoembolization (TACE), radiotherapy, systematic therapy, or supportive therapy. The site of initial recurrence, the causes of mortality, dates of tumor recurrence, death and last follow-up were recorded.

### Study Endpoints and Propensity Score Matching (PSM)

For the purposes of this study, patients with HBV/HCC-HCC and HBV-HCC were compared. HBV/HCV-HCC was defined as pathologically confirmed HCC with serum anti-HCV and HBsAg positivity, while HBV-HCC was defined as pathologically confirmed HCC with serum HBsAg positivity and anti-HCV negativity. The study endpoints were long-term oncological outcomes including overall survival (OS) and recurrence-free survival (RFS). OS was defined as the time from surgery to death from any cause, while RFS was defined as the time from surgery to tumor recurrence or occurrence of a new HCC, or death with evidence of recurrence.

After excluding patients with postoperative 30-day death, patients with HBV/HCV-HCC were matched with patients with HBV-HCC using the propensity score matching (PSM) method as previously described by Rubin and Rosenbaum (16). Covariates entered into the PSM model included age, sex, diabetes mellitus, overweight (BMI > 24kg/m^2^), ASA score, cirrhosis, portal hypertension, Child-Pugh grading, preoperative AST, ALT, and AFP levels, largest tumor size, tumor number, macrovascular and microvascular invasion, satellite nodules, and tumor differentiation. The model was used to provide a one-to-one match between the two groups; details of the matching procedure have been described previously (11, 17, 18).

### Statistical Analysis

Statistical analysis was performed using the IBM SPSS Statistics version 25.0. Continuous variables were expressed as mean ± standard deviations or medians (range) as appropriate after testing for normal distribution using the Kolmogorov-Smirnov test. Categorical variables were reported as numbers (n) and proportions (%). Continuous variables were compared using the Student t test and categorical variables were compared using the Fisher’s exact test or the χ2 test, as appropriate. OS and RFS were calculated by the Kaplan-Meier method and patients with HBV/HCV-HCC were compared with those with HBV-HCC using the log-rank test before and after PSM. Univariable and multivariable Cox proportional hazard regression analyses were performed to identify risk factors associated with OS and RFS after curative liver resection for HCC among patients with HBV/HCV co-infection. All the statistical tests were two-tailed and a *P* value of less than 0.05 was considered statistically significant.

## Results

**Figure 1:**
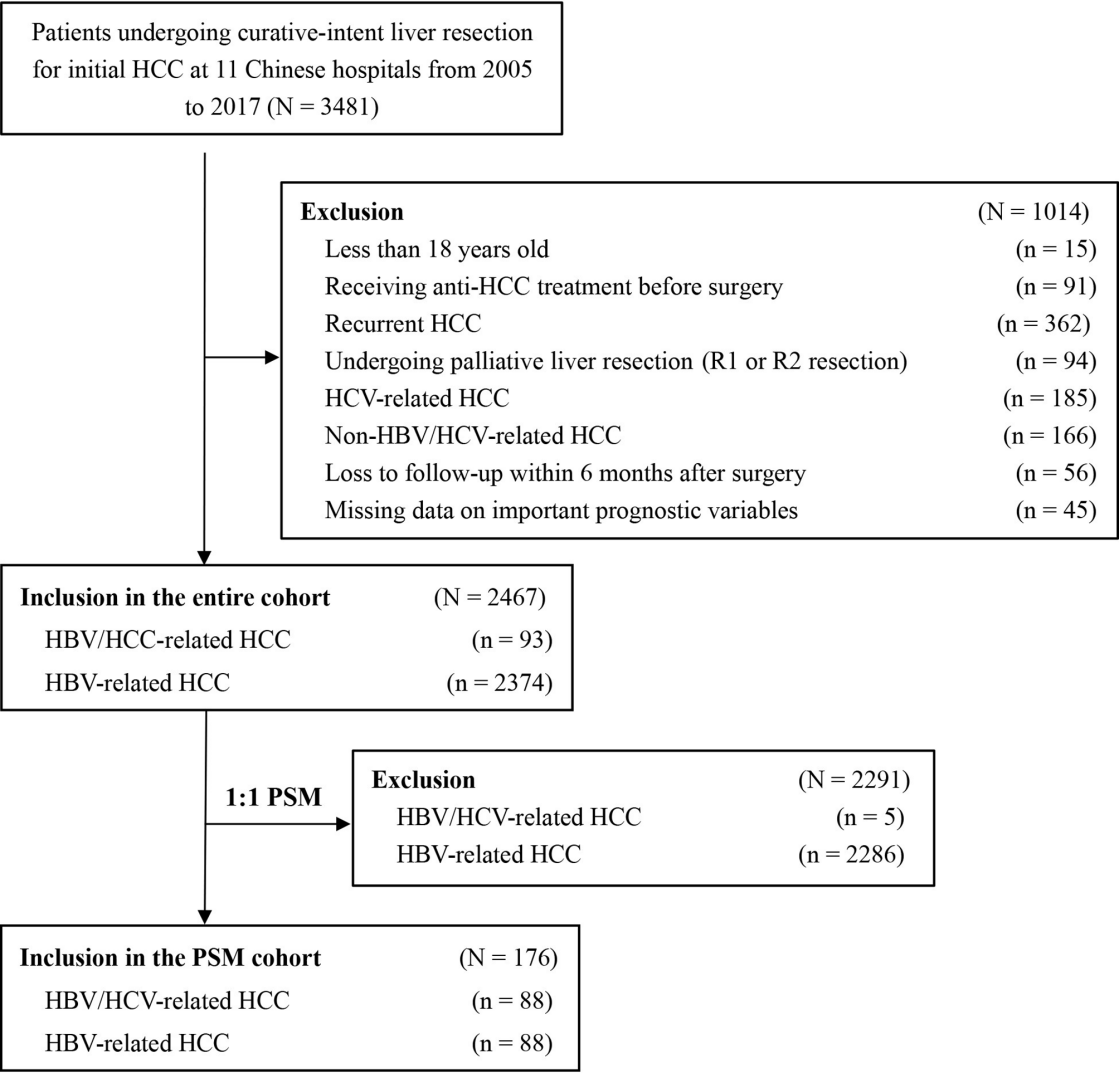
Selection of the study population.

Among the 3,481 patients initially identified, 1014 patients did not fit the inclusion criteria and were excluded. The analytic cohort consisted of 2,467 patients with HBV/HCV-HCC or HBV-HCC who underwent curative liver resection (**Figure 1**). There were 93 patients with HBV/HCV-HCC and 2,374 with HBV-HCC. PSM was used to create 88 matched pairs of patients who had HBV/HCV-HCC or HBV-HCC.

### Comparisons of Clinicopathological Variables and Short-term Outcomes

Patient baseline characteristics, operative variables, and short-term outcomes between patients with HBV/HCV-HCC and HBV-HCC before and after PSM are shown in **Table 1**. Compared with patients who had HBV-HCC, patients with HBV/HCV-HCC were older (Age > 60 years: 44.1% *vs*. 19.0%), more often had cirrhosis (92.5% *vs*. 77.7%) and portal hypertension (43.1% *vs*. 27.3%) (all *P* < 0.05). Patients with HBV/HCV-HCC were less often to have larger tumors (largest tumor size > 5 cm: 31.2% *vs*. 47.7%), multiple tumors (10.8% *vs*. 20.7%) or satellite nodules (12.9% *vs*. 23.1%) (all *P* < 0.05). There were no significant differences in other baseline characteristics and operative variables between the two groups. Patients with HBV/HCV-HCC had higher postoperative mortality and morbidity rates than patients with HBV-HCC (4.3% and 50.5% *vs*. 1.3% and 33.1%, both *P* < 0.05).

**Table 1:** Comparisons of patients’ clinicopathologic characteristics, operative variables, and short-term outcomes between patients with HBV/HCV-HCC and HBV-HCC before and after propensity score matching.

	Before PSM			After PSM		
	HBV-HCC (N=2374)	HBV/HCV-HCC (N=93)	*P* value	HBV-HCC (N=88)	HBV/HCV-HCC (N=88)	*P* value
**Age > 60 years**	451 (19.0)	41 (44.1)	<0.001	35 (39.8)	40 (45.4)	0.446
**Male Sex**	2120 (89.3)	81 (87.1)	0.501	78 (88.6)	78 (88.6)	1.000
**Diabetes mellitus**	131 (5.5)	9 (9.7)	0.089	9 (10.2)	8 (9.1)	0.799
**Overweight (BMI > 24kg/m^2^)**	869 (36.6)	26 (28.0)	0.060	28 (31.8)	24 (27.3)	0.508
**ASA score > 2**	291 (12.3)	11 (11.8)	0.901	8 (9.1)	11 (12.5)	0.466
**Child-Pugh grade B**	235 (9.9)	13 (14.0)	0.199	7 (8.0)	11 (12.5)	0.320
**Cirrhosis**	1844 (77.7)	86 (92.5)	0.001	77 (87.5)	83 (94.3)	0.116
**Portal hypertension**	563 (23.7)	40 (43.1)	<0.001	30 (34.1)	37 (42.0)	0.277
**Preoperative anti-HBV therapy**	906 (38.2)	42 (45.2)	0.174	38 (43.2)	40 (45.4)	0.762
**Preoperative HBV-DNA >10^4^ copies/ml**	1010 (42.5)	32 (34.4)	0.119	34 (38.6)	30 (34.1)	0.531
**Preoperative bilirubin > 17 umol/L**	638 (26.9)	18 (19.4)	0.107	15 (17.0)	17 (19.3)	0.696
**Preoperative albumin < 35 g/L**	278 (11.7)	10 (10.8)	0.778	9 (10.2)	8 (9.1)	0.799
**Preoperative ALT > 40 U/L**	1031 (45.0)	40 (43.9)	0.938	31 (35.2)	37 (42.0)	0.353
**Preoperative AST > 40 U/L**	962 (40.5)	38 (40.9)	0.950	28 (31.8)	36 (40.9)	0.210
**Preoperative AFP > 400 ug/L**	946 (39.8)	33 (35.5)	0.399	31 (35.2)	30 (34.1)	0.874
**Largest tumor size > 5 cm**	1132 (47.7)	29 (31.2)	0.002	23 (26.1)	27 (30.7)	0.504
**Multiple tumors**	492 (20.7)	10 (10.8)	0.019	15 (17.0)	10 (11.4)	0.280
**Macroscopic vascular invasion**	303 (12.8)	13 (14.0)	0.731	11 (12.5)	11 (12.5)	1.000
**Microscopic vascular invasion**	103 (43.4)	31 (33.3)	0.055	24 (27.3)	30 (34.1)	0.327
**Satellite nodules**	548 (23.1)	12 (12.9)	0.022	9 (10.2)	11 (12.5)	0.635
**Poor tumor differentiation**	1490 (62.8)	53 (57.0)	0.259	54 (61.4)	49 (55.7)	0.444
**Incomplete tumor encapsulation**	1348 (56.8)	55 (59.1)	0.652	59 (67.0)	51 (58.0)	0.213
**Intraoperative blood loss > 400 ml**	1007 (42.4)	32 (34.4)	0.125	24 (27.3)	27 (30.7)	0.618
**Intraoperative blood transfusion**	600 (25.3)	22 (23.7)	0.724	12 (13.6)	19 (21.6)	0.166
**Major hepatectomy**	531 (22.4)	17 (18.3)	0.352	20 (22.7)	14 (15.9)	0.252
**Anatomical resection**	736 (31.0)	34 (36.6)	0.257	38 (43.2)	32 (36.4)	0.355
**Resection margin < 1 cm**	1209 (50.9)	48 (51.6)	0.897	55 (62.5)	46 (52.3)	0.170
**Postoperative 30-day mortality**	31 (1.3)	4 (4.3)	0.016	0 (0)	0 (0)	1.000
**Postoperative 90-day mortality**	84 (3.5)	6 (6.4)	0.142	3 (3.4)	2 (2.3)	0.650
**Postoperative 30-day morbidity**	786 (33.1)	47 (50.5)	<0.001	39 (44.3)	42 (47.7)	0.650
** Minor (Clavien-Dindo I-II)**	481 (20.3)	29 (31.2)	0.011	26 (29.5)	28 (31.8)	0.743
** Major (Clavien-Dindo III-V)**	305 (12.8)	18 (19.4)	0.068	13 (14.8)	14 (15.9)	0.834

AFP, Alpha-feto protein; ASA, American Society of Anesthesiologists; AST, Aspartate aminotransferase; ALT, Alanine aminotransferase; BMI, Body mass index; HBV, Hepatitis B virus; HCV, Hepatitis C virus; PSM, Propensity score matching.

After PSM, all the clinicopathological and operative variables were balanced among the two groups of patients with HBV/HCC-HCC and HBV-HCC (all *P* > 0.02). After PSM, postoperative overall morbidity (47.7% *vs*. 44.3%, *P* = 0.650), minor morbidity (31.8% *vs*. 29.5%, *P* = 0.743) and major morbidity (15.9% *vs*. 14.8%, *P* = 0.834) rates were comparable between the two groups.

### Comparisons of Long-term Outcomes

**Figure 2:**
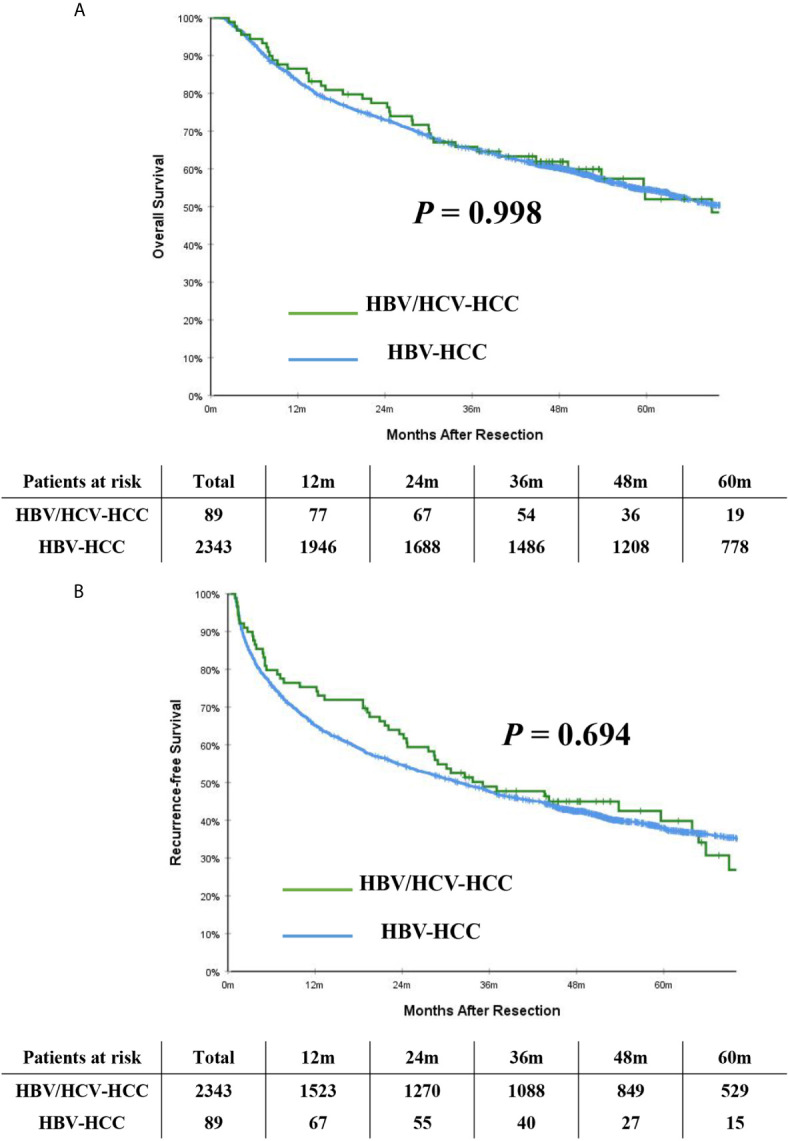
Kaplan-Meier curves of overall survival **(A)** and recurrence-free survival **(B)** between patients with HBV/HCV-HCC and HBV-HCC in the entire cohort.

**Table 2** demonstrates long-term outcomes of patients with HBV/HCV-HCC versus HBV-HCC before and after PSM. With a median follow-up of 59.8 months, mortality and recurrence were observed in 47.2% and 52.8% of patients with HBV/HCV-HCC, respectively; in contrast, mortality and recurrence occurred in 52.4% and 61.9% of patients with HBV-HCC, respectively (*P* = 0.333 and 0.084, respectively). In analyzing the entire cohort, the 5-year OS and RFS rates after curative liver resection among patients with HBV/HCV-HCC were 51.9% and 39.8%, respectively, when compared with 54.5% and 37.9%, respectively in patients with HBV-HCC (*P* = 0.998 and 0.694, respectively) (**Figures 2A, B**).

**Table 2:** Comparisons of long-term outcomes between patients with HBV/HCV-HCC and HBV-HCC before and after propensity score matching.

	Before PSM*			After PSM		
	HBV-HCC (N=2343)	HBV/HCV-HCC (N=89)	*P* value	HBV-HCC (N=88)	HBV/HCV-HCC (N=88)	*P* value
**Recurrence during the follow-up**	1450 (61.9)	47 (52.8)	0.084	23 (26.1)	47 (53.4)	<0.001
**Site of initial recurrence**						
**Intrahepatic**	1094 (46.7)	34 (38.2)	0.115	19 (21.6)	34 (38.6)	0.014
** Extrahepatic**	142 (6.1)	3 (3.4)	0.293	1 (1.1)	3 (3.4)	0.312
** Intra- & Extrahepatic**	214 (9.1)	10 (11.2)	0.501	3 (3.4)	10 (11.4)	0.044
**Mortality during the follow-up**	1228 (52.4)	42 (47.2)	0.333	33 (37.5)	41 (46.6)	0.222
**Cancer-specific mortality**	1089 (46.5)	35 (39.3)	0.184	28 (31.8)	35 (39.8)	0.271
**Non-cancer-specific mortality**	139 (5.9)	7 (7.9)	0.451	5 (5.7)	6 (6.8)	0.755
**OS**						
**1-year OS rate, %**	83.4	86.5	0.998	85.2	86.4	0.081
**3-year OS rate, %**	65.4	65.8		73.7	65.4	
**5-year OS rate, %**	54.5	51.9		63.0	51.1	
**RFS**						
**1-year RFS rate, %**	65.2	75.3	0.694	77.3	75.0	0.037
**3-year RFS rate, %**	47.7	48.9		61.8	48.3	
**5-year RFS rate, %**	37.9	39.8		49.2	38.9	

*Remove the cases of operative death (n = 35).HBV, Hepatitis B virus; HCV, Hepatitis C virus; OS, Overall survival; PSM, Propensity score matching; RFS, Recurrence-free survival.

**Figure 3:**
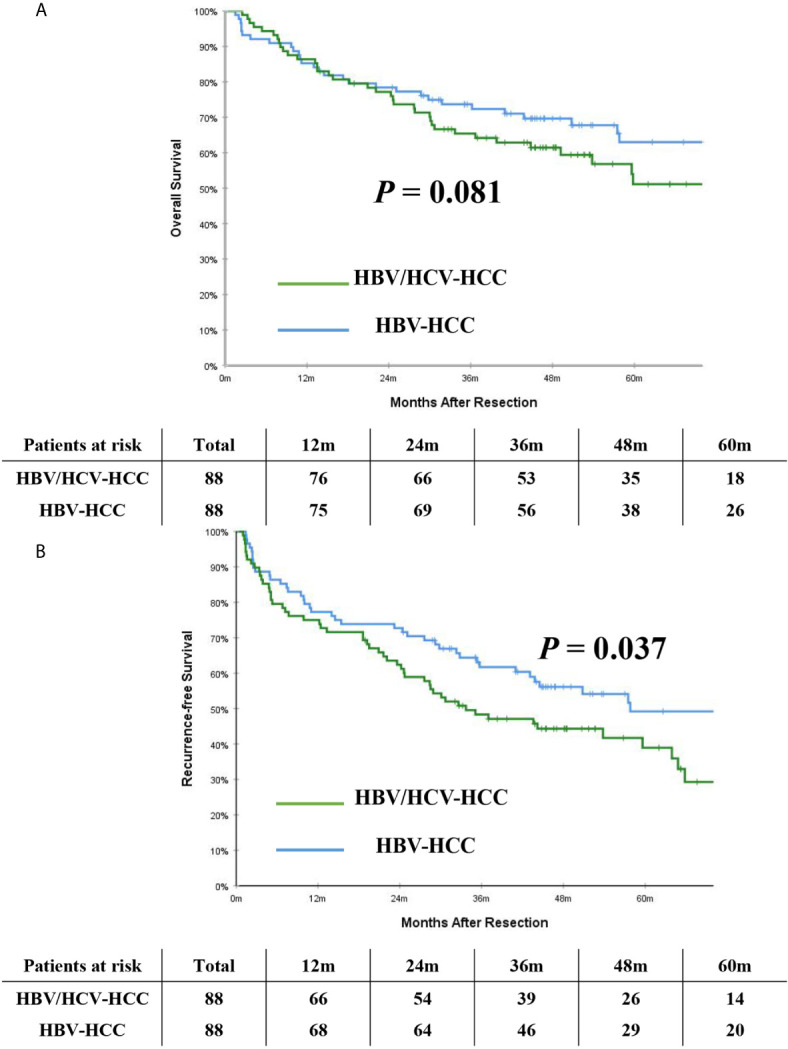
Kaplan-Meier curves of overall survival **(A)** and recurrence-free survival **(B)** between patients with HBV/HCV-HCC and HBV-HCC in the propensity score matching cohort.

After PSM, the incidence of mortality among the 88 patients with HBV/HCV-HCC was still comparable to the matched 88 patients who had HBV-HCC (46.6% *vs.* 37.5%, *P* = 0.222); however, the incidence of recurrence among the HBV/HCV-HCC group was higher than the HBV-HCC group (53.4% *vs.* 26.1%, *P* < 0.001). On PSM, the 5-year OS and RFS rates among patients with HBV/HCV-HCC were 51.1% and 38.9%, which were worse than patients who had HBV-HCC (63.0% and 49.2%, *P*= 0.081 and 0.037, respectively). In the PSM cohort, OS and RFS among patients with HBV/HCV-HCC *versus* patients with HBV-HCC are demonstrated in **Figures 3A, B**.

### Prognostic Analysis for Patients With HBV/HCV-HCC

On multivariable analysis after controlling for competing risk factors (**Table 3**), Child-Pugh grade B, largest tumor size > 5cm, multiple tumors, and macroscopic vascular invasion were independent risk factors associated with poorer OS, while largest tumor size > 5cm, multiple tumors, and macroscopic and microscopic vascular invasion were independent risk factors associated with worse RFS among patients undergoing curative liver resection for HBV/HCV-HCC. Compared with independent risk factors associated with OS and RFS after curative resection for HBV-HCC (**Table 4**), there were some common independent risk factors between patients with HBV-HCV-HCC and patients with HBV-HCC, including largest tumor size > 5cm, multiple tumors, and macroscopic and microscopic vascular invasion.

**Table 3:** Independent prognostic factors associated with overall survival and recurrence-free survival after liver resection for hepatocellular carcinoma among patients with HBV/HCV co-infection by multivariable Cox-regression analysis.

Variables	MV HR (95% CI)	MV *P*
**Independent risks of overall survival**		
Child-Pugh grade B	2.16 (1.04-4.69)	0.044
Largest tumor size > 5 cm	2.20 (1.11-4.36)	0.024
Multiple tumors	3.55 (1.60-7.90)	0.002
Macroscopic vascular invasion	2.80 (1.25-6.29)	0.013
**Independent risks of recurrence-free survival**		
Largest tumor size > 5 cm	2.35 (1.35-4.09)	0.002
Multiple tumors	2.58 (1.22-5.42)	0.013
Macroscopic vascular invasion	2.31 (1.12-4.75)	0.023
Microscopic vascular invasion	2.99 (1.62-5.50)	< 0.001

HBV, Hepatitis B virus; HCV, Hepatitis C virus; CI, Confidence interval; HR, Hazard ratio; MV, Multivariable.

**Table 4:** Independent prognostic factors associated with overall survival and recurrence-free survival after liver resection for hepatocellular carcinoma among patients with only HBV infection by multivariable Cox-regression analysis.

Variables	MV HR (95% CI)	MV *P*
**Independent risks of overall survival**		
Cirrhosis	1.23 (1.06-1.43)	0.008
Preoperative HBV-DNA >10^4^ copies/ml	1.24 (1.10-1.39)	<0.001
Macroscopic vascular invasion	2.51 (2.15-2.95)	<0.001
Microscopic vascular invasion	1.31 (1.14-1.50)	<0.001
Largest tumor size > 5 cm	1.57 (1.36-1.81)	<0.001
Multiple tumors	1.57 (1.36-1.81)	<0.001
Intraoperative blood transfusion	1.40 (1.23-1.61)	<0.001
**Independent risks of recurrence-free survival**		
Satellite nodules	1.60 (1.42-1.81)	<0.001
Largest tumor size > 5 cm	1.38 (1.22-1.55)	<0.001
Multiple tumors	1.54 (1.36-1.74)	<0.001
Macroscopic vascular invasion	2.47 (2.13-2.87)	<0.001
Microscopic vascular invasion	1.27 (1.12-1.43)	<0.001
Resection margin < 1 cm	2.02 (1.81-2.25)	<0.001
Intraoperative blood transfusion	1.20 (1.06-1.36)	0.003

HBV, Hepatitis B virus; CI, Confidence interval; HR, Hazard ratio; MV, Multivariable.

## Discussion

Multiple studies have reported that patients with HBV/HCV co-infection progress faster, have more severe liver disease, and have a higher risk of liver cirrhosis and HCC *versus* patients with HBV or HCV infection alone (6–8, 19–25). Comparisons of clinical patterns and prognosis, including morbidity and mortality, as well as survival and recurrence, after liver resection among patients with HBV/HCV-HCC *versus* patients with HBV-HCC have not been well studied. In the current study, data from a large multicenter observational cohort was utilized to identify patients with co-infection with HBV/HCV-HCC. Of note, patients with HBV/HCV-HCC were older, more often had cirrhosis and portal hypertension, yet less often had larger tumors, multiple tumors, or satellite nodules than patients with HBV-HCC. In the entire cohort, although patients with HBV/HCV-HCC had a reasonably lower postoperative mortality rate and an acceptable postoperative morbidity rate after liver resection, the mortality and morbidity rates were higher than patients with HBV-HCC. After balancing baseline and clinicopathological characteristics using PSM, patients with HBV/HCV-HCC had lower 5-year OS and RFS rates after surgery (51.1% and 38.9%, respectively) *versus* patients with HBV-HCC (63.0% and 49.2%, respectively). In addition, largest tumor size > 5cm, multiple tumors, and macroscopic vascular invasion were independent risk factors associated with worse OS and RFS after liver resection for HBV/HCV-HCC. To our knowledge, this is the first study to evaluate surgical outcomes of liver resection for HBV/HCV-HCC. The results provided useful information that may help inform preoperative planning and postoperative surveillance for patients with HBV/HCV co-infection undergoing liver resection for HCC.

The reasons attributing to the poor short-term and long-term outcomes among patients with HCC who have HBV/HCV co-infection are possibly be related to several factors. Previous studies have suggested a higher risk of developing advanced liver damage among patients with HBV/HCV co-infection *versus* individuals with either infection alone, including the degree of liver fibrosis or cirrhosis, or even the proportion of patients with portal hypertension (26). This may explain the higher postoperative mortality and morbidity rates noted among patients with HBV/HCV-HCC than patients with HBV-HCC. In the current study, almost all (> 90%) patients with HBV/HCV-HCC failed to receive anti-HCV treatment before liver resection due to a low awareness of chronic HCV infection in China where serum HCV test is not routinely obtained until prior to or after surgery. Therefore, although most of the 93 patients had known HBV infection status, less than 30% of patients had a known history of HCV infection until just prior to or after surgery. Furthermore, patients who underwent resection before 2017 were not able to be treated for HCV, as development of effective drugs against HCV infection was after that time. As such, HCC patients with HBV/HCV co-infection may have poorer long-term survival outcomes after liver resection for HCC because many of these patients had not been treated for HCV. The pathogenesis of both HBV and HCV infections is generally immune mediated, yet these viruses have evolved multiple mechanisms to escape immune elimination and continue replicating in an infected host for many years. Multiple carcinogenesis pathways induced by HBV/HCV co-infection, have been postulated to increase severity of liver disease, increase inflammation specifically and increase oncogenicity (8), which can lead to an increased likelihood to develop tumor recurrence or *de novo* tumors than tumors only induced by HBV infection (26). Understanding the common mechanisms of HBV and HCV pathogenesis, including changes of DNA methylation, miRNA expression profile and constitutive activation of many signal transduction pathways will help researchers to focus efforts on therapeutic targets that may be used to develop new treatment approaches for patients with HCC (7, 23, 27, 28).

There are several well-known clinicopathological variables which are associated with long-term prognosis with worse OS and RFS among patients undergoing liver resection for HBV/HCV-HCC. These factors include largest tumor size > 5 cm, multiple tumors, and macroscopic vascular invasion, and patients with these risk factors are particularly need of more intense surveillance and adjuvant therapy following liver resection for HBV/HCV-HCC. Recent guidelines recommend surveillance for tumor recurrence after HCC resection at 4-month intervals for patients with a history of HCV infection. The results of this study suggested standard postoperative anti-HBV and anti-HCV therapies and postoperative surveillance once every 3 months should be recommended for patients undergoing curative liver resection for HBV/HCV-HCC.

The present study had several limitations. The study was retrospective with its inherent proneness to potential biases. There were no unified diagnostic criteria for HBV/HCV-HCC (19), which may result in an underestimation of 40%-50% of unrecognized HCV-infected patients with occult HBV infection [defined as absence of hepatitis B surface antigen and HBV-DNA in serum, but detectable HBV-DNA in liver (6, 8)]. Information of viral genotype, baseline viral load, viral load changes over time, as well as presence or absence of regular anti-hepatitis therapies were also not evaluated. Since patients in this study had resectable tumors with compensated liver function, the data cannot be generalized to all patients with HBV/HCV-HCC. Although the PSM method was used to help balance factors between groups, the relatively small number of patients with HBV/HCV-HCC (n = 93) limited the statistical power. To our knowledge, this study, however, is the first to compare surgical outcomes among patients with HBV/HCC-HCC and patients with HBV-HCC. In addition, patients in the current study were also treated exclusively in China. As such, data from the current study should be externally validated in Western patients to ensure generalizability to a broader population of patients.

## Conclusion

In conclusion, postoperative morbidity and mortality rates, and long-term prognosis among patients with HBV/HCV-HCC were worse compared with patients who underwent resection for HBV-HCC. While several risk factors, including large tumor size, multiple tumors, and macroscopic vascular invasion were already known to be associated with long-term prognosis, the results of this study served to highlight the differences in short- and long-term outcomes among patients undergoing curative liver resection for HBV/HCV-HCC, especially when compared with patients with HBV-HCC.

## Data Availability Statement

The raw data supporting the conclusions of this article will be made available by the authors, without undue reservation.

## Ethics Statement

Ethical review and approval was not required for the study on human participants in accordance with the local legislation and institutional requirements. Written informed consent for participation was not required for this study in accordance with the national legislation and the institutional requirements.

## Author Contributions 

Conception: H-DJ, LL, TY, and D-SH. Study design: H-DJ, LL, CL, HWu, HWa, FS, C-WZ, and TY. Administrative support: FS, D-SH, C-WZ, and TY. Data collection and acquisition: H-DJ, LL, CL, HWu, HWa, Y-JL, Y-HZ, W-MG, X-PF, W-GZ, T-HC, Z-YC, J-HZ, Y-KD, and Q-RX. Data analysis: H-DJ, LL, and TY. Manuscript preparation: H-DJ, LL, CL, HWu, and TY. Critical revision: WL, TP, C-WZ, FS, and D-SH. All authors contributed to the article and approved the submitted version.

## Funding

This work was supported in part by the National Natural Science Foundation of China (No. 81972726, TY). The funding sources had no role in the design and conduct of the study; collection, management, analysis, and interpretation of the data; preparation, review, or approval of the manuscript; and decision to submit the manuscript for publication.

## Conflict of Interest

The authors declare that the research was conducted in the absence of any commercial or financial relationships that could be construed as a potential conflict of interest.

## Publisher’s Note

All claims expressed in this article are solely those of the authors and do not necessarily represent those of their affiliated organizations, or those of the publisher, the editors and the reviewers. Any product that may be evaluated in this article, or claim that may be made by its manufacturer, is not guaranteed or endorsed by the publisher.

## Abbreviations

HCC, Hepatocellular carcinoma; HBV, Hepatitis B virus; HCV, Hepatitis C virus; OS, Overall survival; RFS, Recurrence-free survival; ASA, American Society of Anesthesiologists; AST, Aspartate aminotransferase; ALT, Alanine aminotransferase; BMI, Body mass index; HBsAg, Hepatitis B surface antigen; AFP, Alpha-feto protein; SD, Standard deviation; HR, Hazard ratio; CI, Confidence interval; DAAs, Direct-acting antiviral agents; IFN, Interferon; US, Ultrasonography; PSM, Propensity score matching; MV, Multivariable.
